# Beyond reproduction: Reclaiming the full meaning of sexual and reproductive health

**DOI:** 10.1371/journal.pgph.0007469

**Published:** 2026-11-04

**Authors:** Silvana Matassini Eyzaguirre, Katherine Tumlinson, Angela Muriuki, Nafanta Fadiga

**Affiliations:** 1 Department of Global and Public Health, McGill University, Montreal, Canada; 2 Department of Health Policy and Management, University of North Carolina at Chapel Hill, Chapel Hill, North Carolina, United States of America; 3 Carolina Population Center, University of North Carolina at Chapel Hill, Chapel Hill, North Carolina, United States of America; 4 Consultant, Nairobi, Kenya; 5 Department of Family Medicine, McGill University, Montreal, Quebec, Canada; PLOS: Public Library of Science, UNITED STATES OF AMERICA

Across the world, people with painful menstruation, dyspareunia, or severe perimenopausal symptoms repeatedly hear the same phrase: it's normal. Normal to bleed heavily or for longer than usual. Normal for pain to prevent daily functioning. Normal to lose sexual desire. The problem is not simply that these forms of suffering remain neglected. It is that they have become morally ordinary.

Sexual and reproductive health (SRH) is, by definition [1], a broad field concerned not simply with reproduction, but with sexual wellbeing and the health and functioning of the reproductive system across the life course. Its two constituent concepts are themselves expansive. WHO defines sexual health positively, including physical, emotional, mental, and social wellbeing and the possibility of pleasurable and safe sexual experiences [2]. Reproductive health similarly extends beyond pregnancy and fertility to encompass the health, functions, and processes of the reproductive system across the life course. In this sense, neither sexual health nor reproductive health is reducible to reproduction.

Yet this conceptual breadth has not been matched in practice. Much of the dominant architecture of SRH, its programmes, indicators, and investments, has disproportionately prioritized reproduction and a limited repertoire of measurable reproductive and sexual risks. Sexual health, meanwhile, is commonly operationalized through infection, violence, dysfunction, contraceptive uptake, or reproductive risk, rather than as a dimension of wellbeing in its own right. Meanwhile, dimensions of reproductive health that fall outside reproductive events, including chronic gynecological conditions, sexual pain, and menopause can remain comparatively peripheral. The result is a moral economy in which bodies become most legible to health systems when their needs can be attached to reproductive capacity, reproductive events, or readily countable risks, despite definitions of SRH that promise something considerably broader [Fig 1].

**Fig 1:**
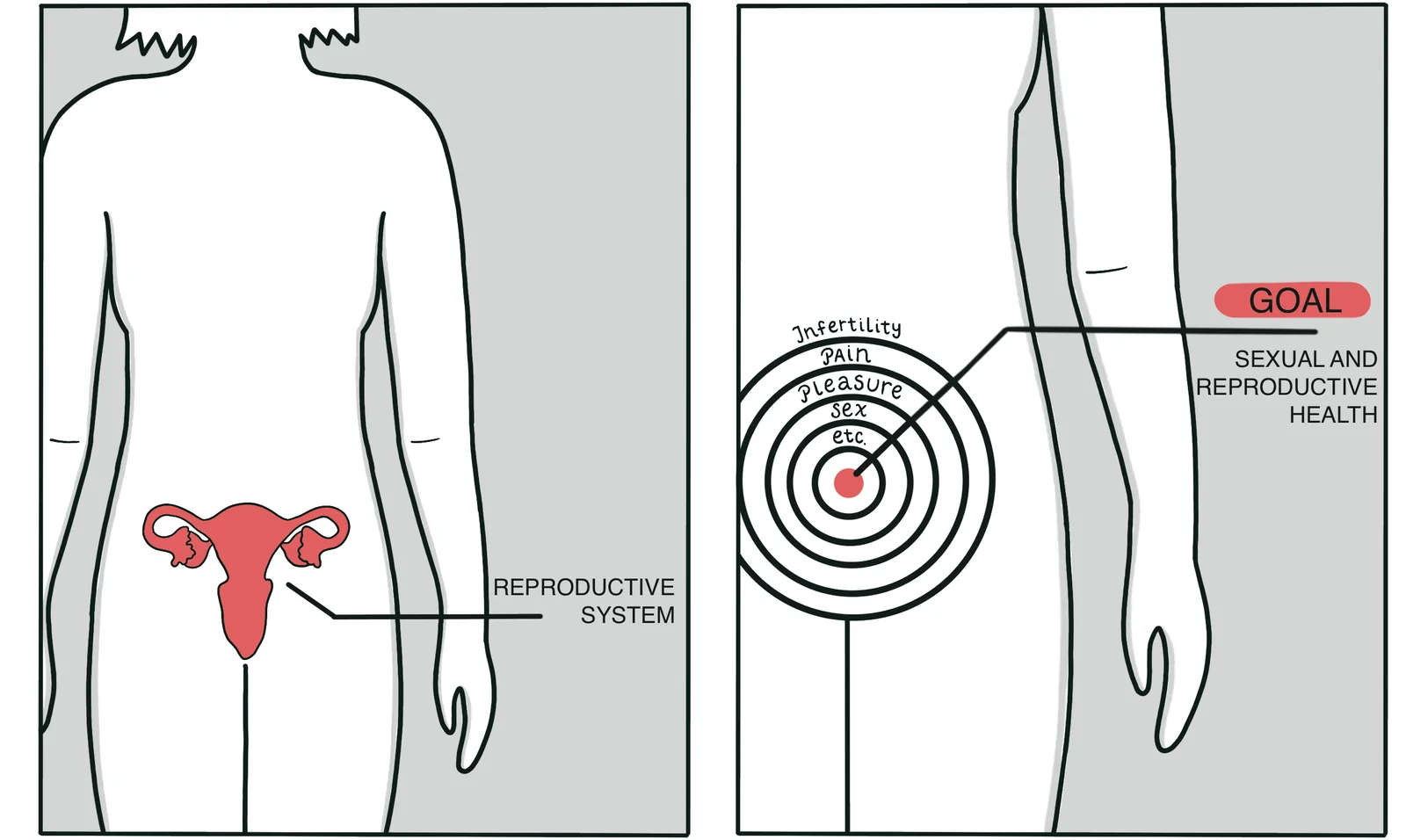
Beyond reproduction: A broader understanding of sexual and reproductive health. Illustrated by Camila Arévalo Harman; conceptualized by Silvana Matassini Eyzaguirre and Camila Arévalo Harman.

This narrowing matters because it shapes which forms of suffering institutions are prepared to recognize and act upon. Pain is particularly revealing in this regard. Across sexual and reproductive health, pain can profoundly shape bodily and sexual wellbeing, relationships, everyday functioning, and quality of life [3]. Yet pain associated with conditions such as endometriosis is frequently normalized or dismissed, contributing to delays in recognition, diagnosis, and treatment [4]. This is especially striking given that sexual health is explicitly defined in terms of wellbeing and the possibility of safe and pleasurable sexual experiences [2]. Taking that definition seriously requires recognizing pain and discomfort as legitimate sexual and reproductive health concerns, regardless of their consequences for reproduction.

Endometriosis and menopause reveal the consequences of this reproductive tunnel vision. Endometriosis affects an estimated 190 million women of reproductive age worldwide and can cause severe menstrual pain, chronic pelvic pain, painful sex, fatigue, and infertility [5]. Yet infertility can become endometriosis’s most institutionally legible consequence prompting action when it threatens reproduction rather than when pain itself diminishes a person's life [5]..

Menopause exposes the opposite boundary. Once reproductive capacity is ending, people disappear from SRH programs altogether. Menopause is neither a disease nor a uniform experience, but symptoms can substantially affect sleep, mental wellbeing, sexual comfort, work, and daily life [6]. It reveals how assumptions about ageing, sexuality, and reproductive value can shape which needs are recognized as legitimate sexual and reproductive health concerns.

These cases also show how an orientation around discrete reproductive events can fragment experiences that people live as connected. Someone with endometriosis may move among primary care, gynecology, fertility services, pain management, and mental health care, while someone seeking menopause care may encounter separate services for sleep, mood, urinary symptoms, cardiovascular risk, or painful sex. When no part of the system assumes responsibility for the whole experience, the work of reconnecting care is shifted onto individuals themselves [7,8].

These omissions are not accidental gaps. They reflect reproductive governance: the political, economic, and institutional processes through which some concerns become measurable and actionable while others remain private, inevitable, or unimportant [9]. These processes are also deeply gendered, reproducing hierarchies of visibility in which women’s health becomes most legible when attached to reproduction, while pain, sexuality, ageing, and everyday wellbeing are more easily normalized or rendered peripheral. The organization of global health around short funding cycles and countable events can reinforce these hierarchies. Contraceptive uptake, births attended, infections treated, and maternal deaths averted are easier to package as programme outputs than relief from chronic pain, dignified ageing, sexual wellbeing, or the capacity to participate fully in social and economic life.

Moving beyond reproduction does not mean displacing pregnancy, contraception, maternal health, or infection prevention from SRH. But reclaiming the broader meaning of SRH also requires attention to the political and economic arrangements that make some forms of suffering more visible and actionable than others. Feminist political economy is particularly useful here, revealing how gendered relations of care, labour, and value shape whose pain is discounted, who absorbs the everyday work of navigating inadequate systems, and which health needs become priorities for states, markets or donors [10]. These perspectives help shift the question from which additional conditions should be included in SRH to the broader institutional logics through which health needs become recognized and valued.

This reframing requires practical change. SRH strategies should treat pain, sexual wellbeing, functional capacity, diagnostic delay, treatment preferences, and quality of life as legitimate outcomes in their own right, rather than only when they affect fertility or contraceptive uptake. Primary care systems should be equipped to recognize menstrual disorders, endometriosis, perimenopause, menopause, and sexual health concerns early, provide appropriate first-line management, and ensure timely referral to specialist care when needed. Research should include adolescents, people not seeking pregnancy, and people beyond conventionally defined reproductive age. Funding should support continuity of care rather than isolated reproductive episodes.

Names shape priorities. “Sexual and reproductive health” should not become shorthand for reproductive risk management. The field should be accountable to the full breadth of its name: sexual wellbeing as an end in itself and reproductive health as a dimension of embodied life that extends beyond reproduction. Moving beyond reproduction therefore does not mean adding an ever-expanding list of conditions to existing agendas. It means reconsidering the institutional boundaries through which some health needs become recognizable, measurable, and actionable while others remain peripheral. Reclaiming the full meaning of SRH means taking seriously what the field has long promised: that sexual and reproductive health matter not only because people reproduce, but because they are fundamental dimensions of human wellbeing.

## References

[1] World Health Organization. Sexual health and its linkages to reproductive health: an operational approach [Internet]. Geneva: World Health Organization; 2017 [cited 2026 Sep 9]. Available from: https://www.who.int/publications/i/item/978924151288

[2] World Health Organization. Defining sexual health. World Health Organization [Internet]. Geneva: World Health Organization; [cited 2026 Aug 4]. Available from: https://www.who.int/teams/sexual-and-reproductive-health-and-research/key-areas-of-work/sexual-health/defining-sexual-health

[3] Ayorinde AA, Macfarlane GJ, Saraswat L, Bhattacharya S. A meta-synthesis of qualitative literature on female chronic pelvic pain for the development of a core outcome set: A systematic review. Int Urogynecol J. 2021;32(5):1187–94. doi: 10.1007/s00192-021-04713-1 33822256 PMC8139940

[4] Robstad N, Paulsen A, Vistad I, Hott AC, Hansen Berg K, Øgård-Repål A, et al. Experiences of pain communication in endometriosis: A meta-synthesis. Acta Obstet Gynecol Scand. 2025;104(1):39–54. doi: 10.1111/aogs.14995 39440568 PMC11683542

[5] Agarwal SK, Chapron C, Giudice LC, Laufer MR, Leyland N, Missmer SA, et al. Clinical diagnosis of endometriosis: A call to action. Am J Obstet Gynecol. 2019;220(4):354.e1-354.e12. doi: 10.1016/j.ajog.2018.12.039 30625295

[6] Hickey M, LaCroix AZ, Doust J, Mishra GD, Sivakami M, Garlick D, et al. An empowerment model for managing menopause. Lancet. 2024;403(10430):947–57. doi: 10.1016/S0140-6736(23)02799-X 38458214

[7] World Health Organization. Framework to implement a life course approach in practice. Geneva: World Health Organization; 2025.

[8] May CR, Eton DT, Boehmer K, Gallacher K, Hunt K, MacDonald S, et al. Rethinking the patient: Using Burden of Treatment Theory to understand the changing dynamics of illness. BMC Health Serv Res. 2014;14:281. doi: 10.1186/1472-6963-14-281 24969758 PMC4080515

[9] Morgan LM, Roberts EFS. Reproductive governance in Latin America. Anthropol Med. 2012;19(2):241–54. doi: 10.1080/13648470.2012.675046 22889430

[10] Gammage S, Kabeer N, van der Meulen Rodgers Y. Voice and agency: Where are we now? Fem Econ. 2016;22(1):1–29. doi: 10.1080/13545701.2015.1101308

